# Spatial distribution and associated factors of non-uptake of DTP3 vaccination among children aged 12–23 months in East Africa: geospatial analysis

**DOI:** 10.1186/s12889-026-28118-1

**Published:** 2026-06-18

**Authors:** Bereket Yilma Beshah, Aschale Wubete Abebe, Biruktawit Lelisa Eticha, Hailemariam Kassahun Desalegn, Tesfaye Deribe Bedada

**Affiliations:** 1 Department of Health Informatics, School of Public Health, College of Medicine and Health Science, Wollo University, Dessie, Ethiopia; 2 Department of Health Informatics, College of Medicine and Health Science, Wachemo University, Hosanna, Ethiopia; 3 Department of Health Informatics, School of Public Health, Institute of Health, Bule Hora University, Bule Hora, Ethiopia

**Keywords:** Spatial distribution, Non-uptake of DTP3 vaccination, East Africa

## Abstract

**Background:**

The diphtheria-tetanus-pertussis (DTP) vaccine is one of the core vaccines provided through the Expanded Program on Immunization (EPI), which is administered to children in a series of three doses to protect against three bacterial diseases. Previous studies have assessed the factors associated with non-uptake of DTP3 vaccination among children. However, studies incorporating geospatial analysis remain limited, particularly in East Africa. Therefore, this study aimed to assess the spatial distribution and associated factors of non-uptake of DTP3 vaccination among children aged 12–23 months in East Africa.

**Methods:**

A total weighted sample of 14,200 children were included from demographic and health survey data of seven East African countries conducted from 2018 to 2023. ArcGIS Pro was used to explore the spatial distribution of non-uptake of DTP3 vaccination. Furthermore, spatial clusters were identified using Kulldorff’s spatial scan statistics. Multiscale geographically weighted regression analysis was done to assess significant associated factors.

**Results:**

The spatial distribution of non-uptake of DTP3 vaccination in East Africa was clustered. SaTScan identified 291 primary spatial clusters, with a relative risk of 3.97 at a p-value of < 0.001, in the central and most of the northern regions of Mozambique. Multiscale geographically weighted regression revealed that no maternal educational background and fewer than four antenatal care visits were positively associated with non-uptake of DTP3 vaccination, whereas health facility delivery was negatively associated with non-uptake of DTP3 vaccination in East Africa.

**Conclusions:**

In East African countries, the high rate of non-uptake of DTP3 vaccination was observed in eastern and northeastern parts of Kenya; central and northern parts of Mozambique; northern and southern parts of Madagascar; and southern and eastern parts of Ethiopia. Therefore, it is recommended to launch community-based educational campaigns and strengthen maternal and child health services in these hotspot areas.

## Background

Childhood vaccination is the most cost-effective public health intervention to reduce child morbidity and mortality from vaccine-preventable diseases [1]. Globally, it has saved over 4 million children’s lives each year since the Expanded Program on Immunization (EPI) was established by the World Health Organization (WHO) in 1974 [2]. Among the vaccines provided through EPI, the diphtheria-tetanus-pertussis (DTP) vaccine is a core vaccine that protects against three bacterial diseases, namely Corynebacterium diphtheriae (diphtheria), Bordetella pertussis (pertussis or whooping cough), and Clostridium tetani (tetanus)M [3]. The DTP vaccine is administered to a child in a series of three doses; the first dose of DTP is administered at 6 weeks of age, the second dose at 10 weeks of age, and the third dose at 16 weeks of age [4]. Thus, three doses of DTP vaccination coverage is globally recognized as one of the key indicators to measure the overall immunization program performance [5].

WHO, in collaboration with partners, consistently contributes to improve the global routine vaccination coverage and sets a global target of achieving a minimum of 90% DTP3 coverage under the Immunization Agenda 2030 (IA2030) [6]. However, according to WHO reports in 2024, only about 85% of infants globally received the three doses of DTP vaccine, leaving nearly 19.9 million infants unvaccinated for DTP3 [7]. The coverage of DTP3 vaccination significantly varied across regions, with higher coverage (almost 92%) observed in the region of Europe and central Asia, and south Asia, while lower coverage (nearly 76%) was observed in Africa [8, 9]. In Eastern and southern Africa, the coverage of DTP3 vaccination was low (79%) [8], which remains substantially below the global target of 90%. Such suboptimal DTP3 vaccination coverage still threatens progress toward the Sustainable Development Goals (SDG) and has a serious implication for persistence of vaccine-preventable diseases [10].

Non-uptake of DTP3 vaccination significantly increases the risk of developing diphtheria, tetanus, and pertussis disease. These infectious diseases can lead to severe illness, disability and even death in children [11]. Diphtheria infection causes myocarditis, neuritis, and airway obstruction and accounts for approximately 20% of deaths in children under five years of age [12]. Tetanus infection, caused by the bacterium Clostridium tetani, leads to a severe complication in children such as muscular spasm and rigidity, and autonomic nervous system dysfunction [13, 14]. pertussis or whooping cough causes a severe complication such as apnea, pneumonia, encephalopathy, and seizures and sudden death in unvaccinated or incompletely vaccinated infants [15, 16].

Previous studies identified that non-uptake of DTP3 vaccination among children was associated with several factors, such as mother’s educational status [17–19], paternal educational status [18, 19], mother’s working status [17, 20], maternal age [19, 21], parity [17], wealth index [17, 19, 22], place of delivery [18, 19, 21], number of antenatal care (ANC) visits [23, 24], and access to media [20]. However, most existing studies focus on global regression models, which overlook local variations and limit the ability to identify high-risk areas of non-uptake of DTP3 vaccination. Furthermore, studies using cross-national datasets are limited, even though such datasets enable comparisons across countries. Therefore, to address these gaps, this study applied geospatial analysis to assess the spatial distribution of non-uptake of DTP3 vaccination and its spatially varying determinants among children aged 12–23 months in East Africa, using the most recent Demographic and Health Survey (DHS) data collected between 2018 and 2023.

## Methods and materials

### Study area and design

Geographically, East Africa is one of the sub-regions of Africa, which extends to the easternmost parts of the continent [25]. According to United Nations statistics, nineteen countries (Burundi, Comoros, Djibouti, Ethiopia, Eritrea, Kenya, Madagascar, Malawi, Mauritius, Mozambique, Rwanda, Seychelles, Somalia, Tanzania, Uganda, Zambia, South Sudan, Zimbabwe, and Sudan) are categorized as the East Africa sub-region [26]. From these countries, five countries (Djibouti, Somalia, South Sudan, Seychelles, and Mauritius) do not have DHS datasets. Although fourteen countries have DHS datasets, one (Eritrea) has restricted DHS data, and six countries (Sudan, Comoros, Malawi, Burundi, Uganda, and Zimbabwe) have DHS datasets conducted before 2018. Therefore, this study included seven East African countries (Kenya, Tanzania, Mozambique, Madagascar, Rwanda, Zambia, and Ethiopia) which had recent DHS data conducted between 2018 and 2023 to be more representative for East Africa. The DHS survey employed a community-based cross-sectional study design across all countries.

### Sampling procedure

The DHS program has employed a two-stage stratified cluster sampling procedure to select representative samples from the most recent census sampling frame. Samples of DHS were stratified by geographic region of the country. Furthermore, the regions of the country were stratified into urban and rural areas. Within each stratum, the allocation of households per enumeration areas (EAs) were specified. In the first stage, samples of EAs (clusters) were randomly selected with probability proportional to size for each stratum. Then, in the second stage, a fixed number of dwellings/households were selected by equal probability systematic sampling in the selected EAs [27]. The detailed sampling procedure is available on the link www.dhsprogram.com.

### Sample size determination

For this study, the Kids record (KR) dataset of seven East African countries was used to determine the sample. Thus, A total weighted sample of 14,200 children aged 12–23 months were included in this study (Table 1).

**Table 1:** Sample size distribution across each East African countries with their respective DHS year, 2018–2023

East African countries	DHS survey year	Weighted sample
Ethiopia	2019	1028
Kenya	2022	3324
Madagascar	2021	2337
Mozambique	2022	1807
Rwanda	2019	1633
Tanzania	2022	2180
Zambia	2018	1891
Total weighted sample		14200

### Study variables and operational definitions

#### Outcome variable

The outcome variable was non-uptake of DTP3 vaccination. This variable was measured in the DHS Kids record dataset as ‘received DTP3’. For this study, children aged 12–23 months who received the DTP3 vaccine were classified as having DTP3 uptake, while those children who did not receive the DTP3 vaccine were classified as having non-uptake of DTP3. For spatial analysis, this variable was aggregated to the cluster level as the proportion of children who did not receive DTP3 vaccine within each DHS cluster. Therefore, this proportion was treated as a continuous variable.

#### Independent variables

The independent variables were maternal age, marital status (labeled as single, married, divorced, and widowed), maternal education (no education, primary, secondary, and higher education), partner’s education, maternal occupation (labeled as working and not working), wealth index (labeled as poor, middle, and rich), parity, frequency of Watching TV, frequency of reading newspaper/magazine, frequency of listening radio, and internet use, number of ANC visits, health facility distance, and place of delivery.

#### Operational definition

Non-uptake of DTP3 vaccination: operationally defined by WHO and Gavi as children aged 12–23 months who did not receive the third dose of DTP vaccine [1, 28].

### Data management and statistical analysis

The datasets of seven East African countries were downloaded in the standard recode format from the DHS program and imported into STATA V.17 for cleaning and preparation prior to analysis. Each country’s data was checked for completeness. Variables were recoded as needed and standardized to provide consistency in variable nomenclature, categories, and coding. DHS survey sampling weight was done to adjust for differences in probability of selection and interview, which can occur due to survey design or chance. Specifically, the DHS individual sampling weight (v005) was normalized by dividing by 1,000,000 prior to spatial analysis. Weighted estimates were computed at the DHS cluster level for each country and were incorporated into the spatial regression to ensure population representativeness and reduce potential bias arising from unequal sampling probabilities. After preparation, the individual country datasets were appended to create one dataset. Descriptive statistics were employed to summarize the distribution of DTP3 vaccination status across different background characteristics using frequencies and percentages. For spatial analysis, the outcome and independent variables were aggregated to the cluster level by computing proportions within each DHS cluster. Then, the appended dataset was exported to Microsoft Excel and saved in CSV format, which is compatible with ArcGIS Pro and SaTScan software.

### Spatial autocorrelation (Global Moran’s I) statistic

Spatial autocorrelation is used for assessing whether non-uptake of DTP3 vaccination among children aged 12–23 months is dispersed, clustered, or randomly distributed across East Africa. Moran’s I values range from − 1 to + 1, where Moran’s I value close to -1 indicates non-uptake of DTP3 vaccination is dispersed across East Africa; Moran’s I value close to + 1 shows non-uptake of DTP3 vaccination is clustered; and Moran’s I value close to zero shows non-uptake of DTP3 vaccination among children aged 12–23 months is randomly distributed across East Africa. A statistically significant Moran’s I index (*p* < 0.05) leads to the rejection of the null hypothesis [29].

### Hot spot analysis (the Getis-Ord Gi *) statistic

The Getis-Ord Gi* is used to determine the area of non-uptake of DTP3 vaccination among children aged 12–23 months with hotspot and coldspot values found in East Africa. Clusters with the resultant z-scores of greater than 1.96 and p-values of less than 0.05 were defined as hotspots (high-value clusters) of DTP3 vaccine non-uptake, whereas clusters with the resultant z-scores of less than − 1.96 and p-values of less than 0.05 were defined as coldspots (low-value clusters) of DTP3 vaccine non-uptake [30].

### Spatial interpolation

Spatial interpolation is used to predict non-uptake of DTP3 vaccination among children aged 12–23 months for unsampled areas using the data of sampled areas. Deterministic and geostatistical methods were compared using root mean square error (RMSE) and mean predicted error (ME) parameters to determine the best-fitted interpolation technique for non-uptake of DTP3 vaccination across East Africa. The lowest value was selected because it indicated that the predicted values were close to the actual (observed) values.

### Spatial scan statistics

Spatial scan statistics were applied to determine the geographical location of statistically significant spatial windows for non-uptake of DTP3 vaccination among children aged 12–23 months using SaTScan version 10.2.5 software [31]. Kuldorff’s Bernoulli-based spatial scan statistics were applied to identify purely spatial clusters of non-uptake of DTP3 vaccination. Children aged 12–23 months who did not receive the DTP3 vaccination were classified as cases, and those who received the DTP3 vaccination were classified as controls. The maximum spatial cluster size was set to 25% of the population at risk to enhance detection of localized clusters and to avoid identification of overly large clusters. Moreover, log likelihood ratio and p-value results were used to determine whether the cluster was statistically significant or not. The scanning window with the highest log likelihood ratio was identified as the most likely (primary) cluster. Finally, the result of spatial scan statistics was exported to ArcGIS pro software to map the statistically significant clusters and their associated attributes.

### Spatial regression analysis

#### Exploratory regression

Exploratory regression tool was used to evaluate the possible combinations of the explanatory variables to find out the best models which pass all the necessary Ordinary least square regression diagnostics parameters. It tests all combinations explanatory variables for their completeness, redundancy, bias, significance and performance. Exploratory regression in ArcGIS is not only looking for Models with high Adjusted R^2^ values but also it has a similar function like Stepwise regression which found in many statistical software.

#### Ordinary least square (OLS) model

OLS is a starting point for all spatial regression analysis, which provides a single regression equation to model variables within the entire study area. The assumption of OLS was checked to ensure the reliability and validity of the model result. The Jarque-Bera statistic was used to assess whether the residuals are normally distributed or not. Multicollinearity was checked to remove redundancy among predictor variables using the Variance Inflation Factor (VIF). The Koenker (BP) statistic was used to determine whether the explanatory variables in the model had a consistent relationship to the dependent variable both in geographic space and in data space. The statistically significant Koenker (BP) statistic shows that the relationships are not consistent. Robust probability (*p* < 0.05) was used to get statistically significant predictor variables with their coefficients.

#### Multiscale geographically weighted regression (MGWR) model

Geographical weighted regression (GWR) is a local spatial regression model that allow the non-stationary relationship between the dependent variable and explanatory variables. Thus, GWR constructs a separate OLS equation which combines the dependent and explanatory variables for every location falling within the bandwidth of each target location [32]. However, GWR considers that the spatial scale is constant over spaces, which may lead to biased results by overestimating and underestimating local effects of each independent variable on the dependent variable. To overcome the limitation of constant spatial scale, MGWR allows the non-stationary relationship between the dependent variable and independent variables at different spatial scale.

MGWR model can be presented according to the following formula:

$$\mathrm{Y}_\mathrm{i}=\sum\nolimits_{j=1}^{k}\,\upbeta_{\mathrm{bwj}}(\mathrm{u}_{\mathrm{i}},\mathrm{v}_{\mathrm{i}})\mathrm{X}_{\mathrm{ij}}+\mathrm{e}_{\mathrm{i}}$$

Where: Y_i_ = non-uptake of DTP3 vaccination at i^th^ point,

(ui, v𝑖) = geographical coordinates at i^th^ point,

β_bwj_ = local coefficient for j^th^ independent variable, estimated using variable specific bandwidth.

𝑋_ij_= the j^th^ independent variable,

K= number of independent variables,

e_i_=standard error.

#### Model evaluation

Model performance was compared using the Akaike Information Criteria (AIC) and adjusted R-squared. The best fitted model for local parameter estimates was a model with the lower AICc value and a higher adjusted R-squared value.

## Results

### Descriptive statistics

This study included a total of 14,200 children aged 12–23 months in the selected East African countries. In terms of the age distribution, the mean age of women was 28.11. The largest proportion of children (26.85%) belonged to women aged 20–24 years, followed by 25.58% of children who were born to women aged 25–29 years. With respect to women’s wealth index, the largest proportion of children were born to women from a poor wealth index (44.93%), followed by those who were born to women from a rich wealth index (36.74%). Regarding women’s educational attainment, nearly half of children (47.96%) were born to women with primary education, as well as about one-fifth of children (18.64%) were born to women with no education. In terms of place of delivery, almost three-quarters of children (74.1) were delivered in health facilities. Regarding the number of ANC visits, approximately three-fifths of children (60.88%) were born to women who attended four or more visits (Table 2).

**Table 2:** Distribution of non-uptake of DTP3 vaccination among children aged 12–23 months by selected characteristics in East Africa

Variables	Weighted frequency	DTP3 vaccination status	
		Uptake of DTP3 vaccination	Non-uptake of DTP3 vaccination
Maternal age			
15–19	1230 (8.66%)	895	335
20–24	3813 (26.85%)	3010	803
25–29	3633 (25.58%)	2958	675
30–34	2630 (18.52%)	2230	400
35–39	1911 (13.46%)	1589	322
40–44	805 (5.67%)	662	143
45–49	178 (1.26%)	127	51
Wealth index			
Poor	6380 (44.93%)	4746	1634
Middle	2604 (18.34%)	2130	474
Rich	5216 (36.74%)	4595	621
Women’s educational status			
No education	2647 (18.64%)	1647	1000
Primary education	6811 (47.96%)	5559	1252
Secondary education	3857 (27.17%)	3449	408
Higher education	884 (6.23%)	816	68
Maternal working status			
Not working	4928 (34.70%)	3770	1158
Working	9272 (65.30%)	7701	1571
Marital status of women			
Single	1174 (8.3%)	1032	142
Married	11,737 (82.7%)	9464	2273
Widowed	149 (1%)	117	32
Divorced	1140 (8%)	858	282
Partner’s level of education			
No education	1857 (17.3%)	1154	703
Primary education	4831 (45.1%)	4047	784
Secondary education	3044 (28.4%)	2703	341
Higher education	993 (9.3%)	929	64
Parity			
Primiparous	3472 (24.4%)	2925	547
Multiparous	7387 (52.1%)	5982	1404
Grand multiparous	3341 (23.5%)	2564	777
Place of delivery			
Home delivery	3680 (25.9%)	2220	1460
Health facility delivery	10,518 (74.1%)	9250	1268
Maternal tetanus vaccination status during pregnancy			
Received	9646 (76.6%)	8333	1313
Not received	2948 (23.4%)	2041	907
Frequency of watching television			
Not at all	8122 (61.7%)	6265	1857
At least once a week	5050 (38.3%)	4580	470
Frequency of reading newspaper or magazine			
Not all	11,224 (85.2%)	9044	2180
At least once a week	1948 (14.8%)	1801	147
Frequency of listening to radio			
Not all	5750 (43.7%)	4168	1582
At least once a week	7422 (56.3%)	6677	745
Internet use			
Never	10,629 (80.7%)	8516	2113
At least once a week	2543 (19.3%)	2329	214
Number of ANC visit			
< 4 times	5554 (39.22%)	3975	1579
>=4 times	8646 (60.88%)	7497	1149
Health facility distance			
Big problem	3892 (33.6%)	2874	1018
Not a big problem	7684 (66.4%)	6544	1140

Out of the 14,200 children, 2729 (19.21%) children did not receive the third dose of the DTP vaccine. Furthermore, the distribution of non-uptake of DTP3 vaccination among children aged 12–23 months varied across the different characteristics. The percentage of non-uptake of DTP3 vaccination was 60.9% among children born to women with no education and 16.22% among children born to women with primary education. Among children delivered at home, two-thirds of children (65.7%) had non-uptake of the DTP3 vaccination. Among children whose mothers had fewer than four ANC visits, there was a 28.4% non-uptake of the DTP3 vaccination (Table 2).

### Spatial analysis result

#### Spatial autocorrelation of non-uptake of DTP3 vaccination among children

**Fig. 1:**
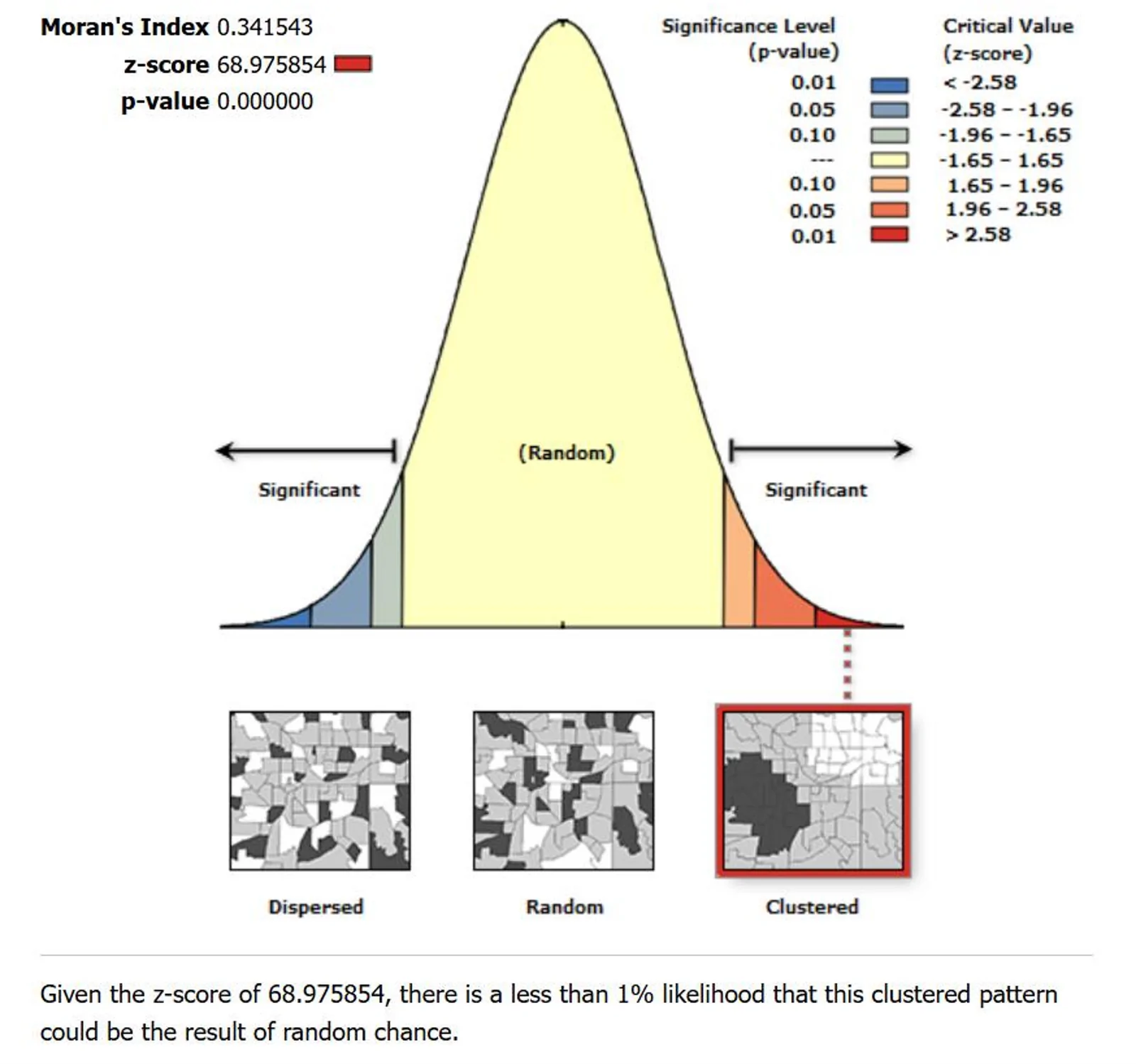
Spatial autocorrelation of non-uptake of DTP3 vaccination among children aged 12–23 months in East Africa

The spatial distribution of non-uptake of DTP3 vaccination among children aged 12–23 months in East Africa was clustered with Global Moran’s I value of 0.3415 (p value < 0.01) and a Z score of 68.976. This result showed that there is less than a 1% probability that the observed clustering of non-uptake of DTP3 vaccination among children aged 12–23 months in East Africa could be the result of random chance (Fig. 1).

#### Hotspot analysis (the Getis-Ord Gi *)

**Fig. 2:**
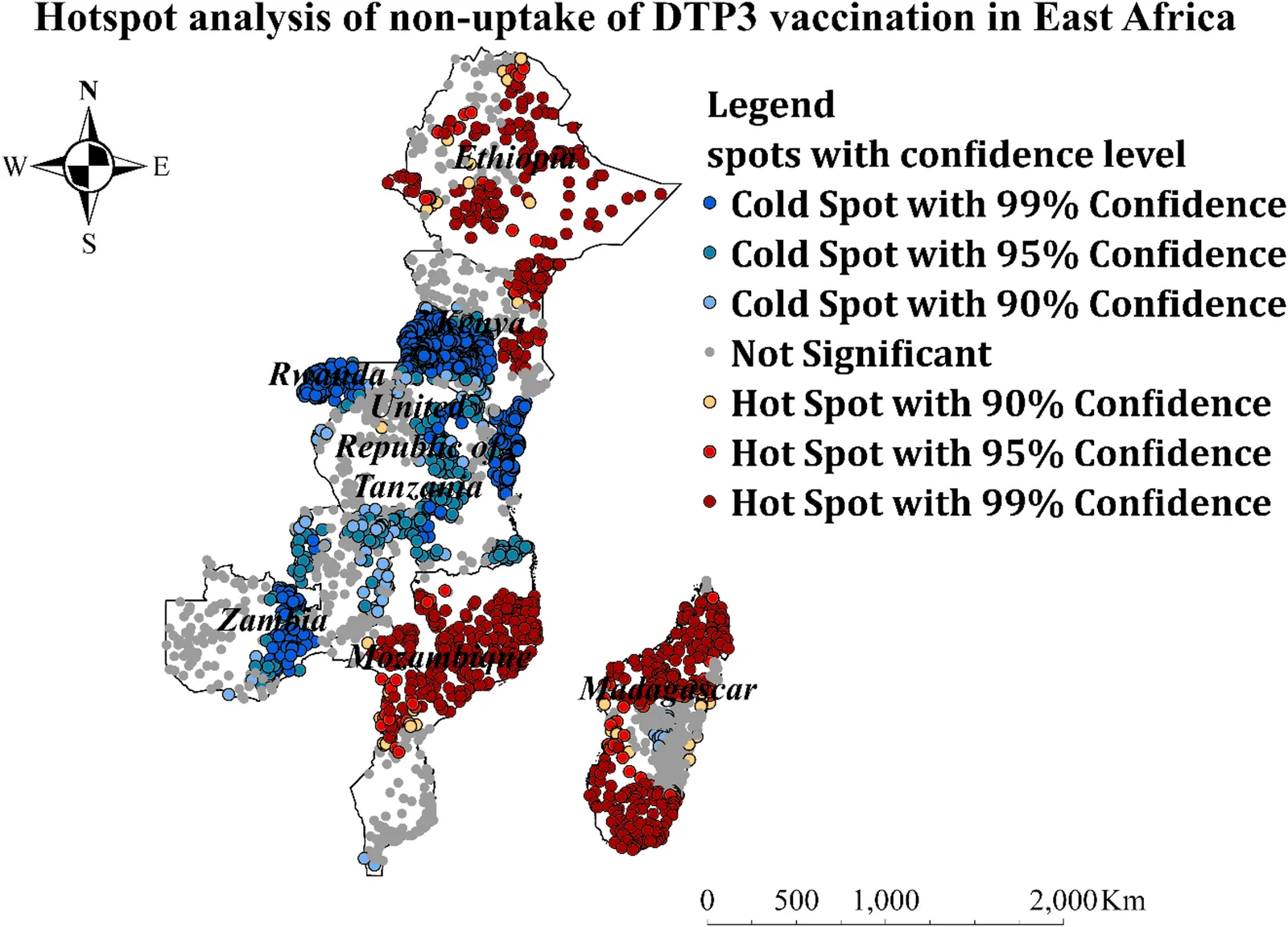
Hotspot analysis of non-uptake of DTP3 vaccination among children aged 12–23 months in East Africa

Hotspot analysis identified the hotspot and coldspot areas of non-uptake of DTP3 vaccination among children aged 12–23 months in East Africa with its confidence level. Statistically significant hotspot areas of non-uptake of DTP3 vaccination with a 95% confidence level were observed in eastern and northeastern parts of Kenya; central and northern parts of Mozambique; northern and southern parts of Madagascar; and southern and eastern parts of Ethiopia. On the other hand, coldspot areas of non-uptake of DTP3 vaccination were detected in the southern parts of Kenya; Rwanda; the central and eastern parts of Tanzania; and the central regions of Zambia (Fig. 2).

#### Spatial interpolation

Ordinary kriging interpolation method was appropriately fitted for pridicting non-uptake of DTP3 vaccination among children aged 12–23 months in unobserved areas of East Africa since its mean error value (0.002733) is the closest to zero, and its root-mean-square error value (0.244063) is the lowest compared to others interpolation method (Table 3).

**Table 3:** Comparison of different interpolation methods for predicting non-uptake of DTP3 vaccination among children aged 12–23 months

Interpolation method	Performance metrics	
	Mean error(ME)	Root mean square error(RMSE)
Deterministic methods		
Inverse distance weighted	-0.008251	0.154019
Radial basis functions	-0.003781	0.246539
Geostatistical Methods		
Ordinary kriging	0.002733	0.244063
Simple kriging	-0.027583	0.246755
Indicator Kriging	0.001881	0.402561
Disjunctive kriging	-0.029826	0.246809

**Fig. 3:**
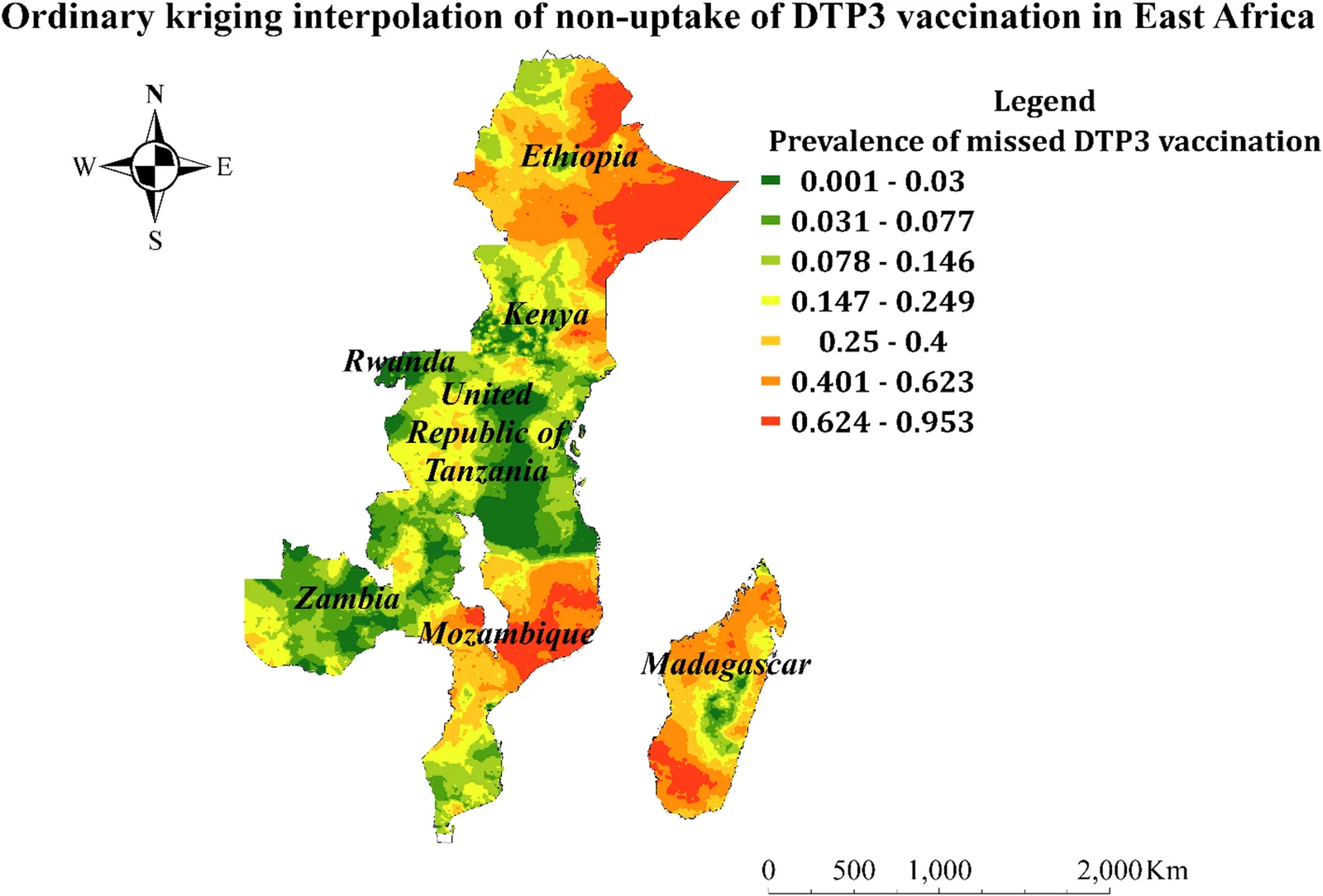
Ordinary Kriging interpolation of non-uptake of DTP3 vaccination in East Africa

According to the ordinary kriging interpolation, the level of non-uptake of DTP3 vaccination among children aged 12–23 months gets higher when the range of color change from green to red. A high risk of non-uptake of DTP3 vaccination was observed in eastern, southern and most western parts of Ethiopia; eastern parts of Kenya; some western parts of Tanzania; northern and central parts of Mozambique; and southern and northern parts of Madagascar. While, a low risk of non-uptake of DTP3 vaccination was observed in central and northern parts of Ethiopia; western parts of Kenya; Rwanda; central and southern parts of Tanzania; Zambia; southern parts of Mozambique; and central Madagascar (Fig. 3).

#### Spatial SaTScan analysis

The result of spatial kulldorff’s scan analysis identified 17 spatial clusters. Out of 17 clusters, four clusters were statistically significant at p value < 0.05. The primary (most likely) spatial cluster which filled in red color was found in the central and most of the northern regions of Mozambique. This spatial window was located at 16.0089° South and 37.0916° East with a radius of 461.479 km, a log-likelihood ratio (LLR) of 597.68, and a relative risk of 3.97 at a p-value of < 0.001. The relative risk of 3.97 showed that children aged 12–23 months within this red spatial window had a 3.97 times higher risk of non-uptake of DTP3 vaccination than children outside this window.   

**Fig. 4:**
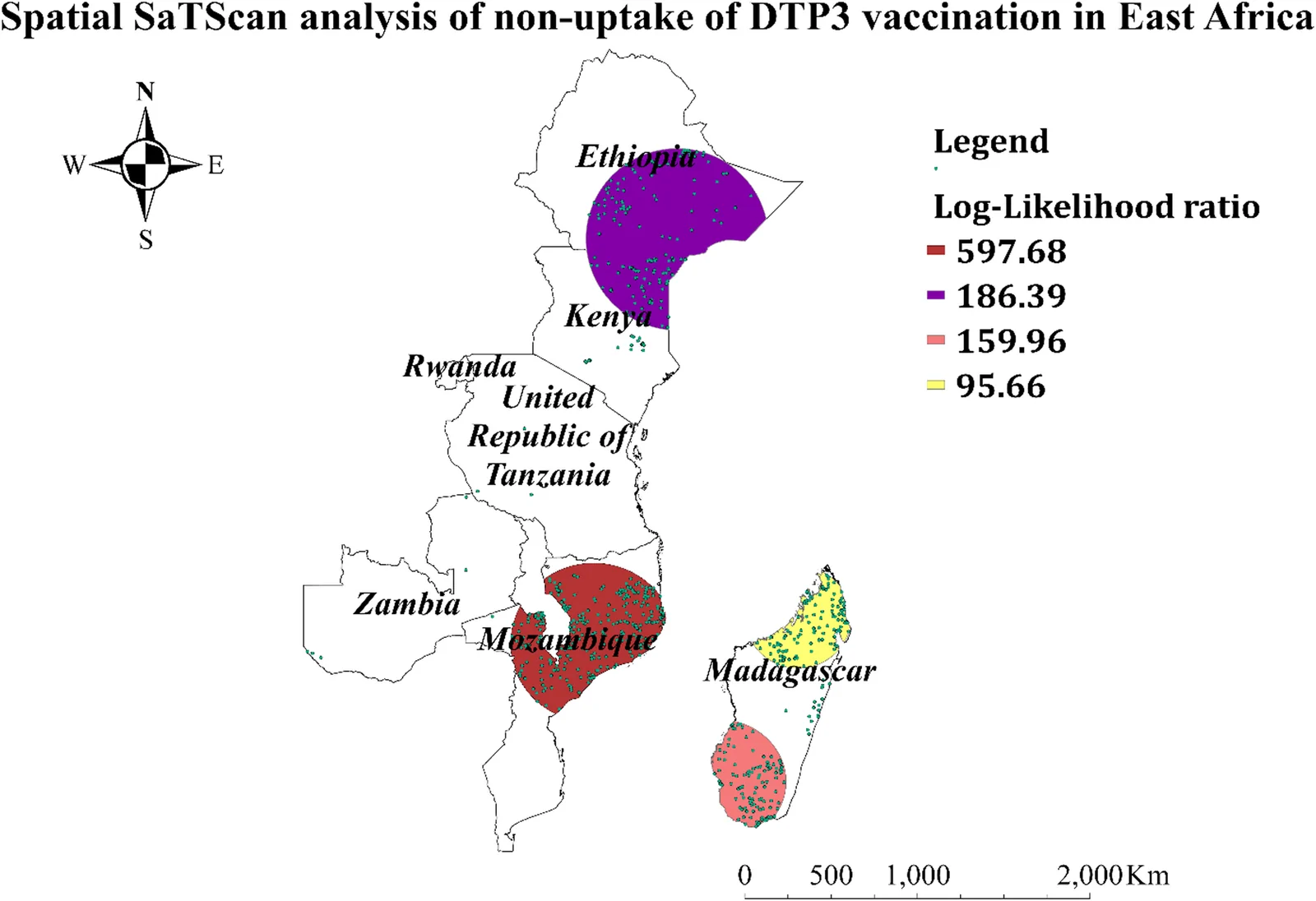
Spatial SaTScan analysis of non-uptake of DTP3 vaccination in East Africa

In addition, three significant spatial clusters were identified as secondary spatial cluster. The spatial window that was filled with purple color was located at 4.996° North and 41.4407° East with a radius of 525.78 km, which covered the most eastern and southern regions of Ethiopia and the northeastern regions of Kenya. The log-likelihood ratio (LLR) and relative risk of this spatial window were 186.39 and 3.12, respectively. The relative risk of 3.12 indicated that children aged 12–23 months within this window had a 3.12 times higher risk of non-uptake of DTP3 vaccination than children outside this window. The orange spatial window, which covered most regions of southwestern Madagascar, was specifically located at 23.203° South and 43.62° East with a radius of 355.04 km, a log-likelihood ratio (LLR) of 159.96, and a relative risk of 3.03 at a p-value of < 0.001. This relative risk showed that children aged 12–23 months within this orange spatial window had a 3.03 times higher risk of non-uptake of DTP3 vaccination than children outside this window. The yellow spatial window, which covered northern parts of Madagascar, was located at 14.666° South and 47.736° East with a radius of 295.36 km, a log-likelihood ratio (LLR) of 95.66, and a relative risk of 2.61 at a p-value of < 0.001. The relative risk of 2.61 indicated that children aged 12–23 months within the yellow spatial window had a 2.61 times higher risk of non-uptake of DTP3 vaccination than children outside this window (Fig. 4 and Table 4).

**Table 4:** Statistically significant spatial clusters of non-uptake of DTP3 vaccination among children aged 12–23 months in East Africa

Types of clusters	# cluster location	Total # case	Total # population	LLR	RR	RADIUS	*p*-value
Primary cluster	291	780	1299	597.68	3.97	461.47	< 0.001
Secondary cluster 1	205	304	548	186.39	3.12	525.76	< 0.001
Secondary cluster 2	111	273	503	159.96	3.03	355.04	< 0.001
Secondary cluster 3	130	212	444	95.66	2.61	295.36	< 0.001

### Spatial regression analysis of non-uptake of DTP3 vaccination

The exploratory regression tool identified a combination of three explanatory variables from a total of fifteen variables based on highest adjusted R-squared and lowest corrected Akaike Information Criterion (AICc). These variables were women with no educational background, women who delivered in a health facility, and women with a history of fewer than four ANC visits. these variables were also used in OLS and MGWR models. The final OLS model result explained 22.5% of the variance in non-uptake of DTP3 vaccination among children aged 12–23 month. Furthermore, the results showed that women with no educational background and women with a history of fewer than four ANC visits were positively associated with non-uptake of DTP3 vaccination among children aged 12–23 month. In contrast, women who delivered in a health facility was negatively associated with non-uptake of DTP3 immunization (Table 5).

**Table 5:** OLS model summary and diagnostics of non-uptake of DTP3 vaccination among children aged 12-23 month in East Africa

Variables	coefficient	Standard Error	probability	Robust probability	VIF
Intercept	0.285631	0.013101	<0.001	<0.001	------
Women with no educational background	0.230209	0.014473	<0.001	<0.001	1.2395
Women who delivered in health facilities	− 0.241860	0.012758	<0.001	<0.001	1.2833
Women with a history of fewer than four ANC visits	0.082416	0.011574	<0.001	<0.001	1.11889

OLS Diagnostics					
Diagnostics criteria	Magnitude		p-value		
AICc	677.558		-------		
Multiple R-Squared	0.2266		-------		
Adjusted R-Squared	0.2251		-------		
Joint F-Statistic	438.3430		<0.001		
Joint Wald Statistic	824.1319		<0.001		
Koenker (BP) Statistic	249.176		<0.001		
Jarque-Bera Statistic	2556.0817		<0.001		

*NB: AICc* Akaike’s Information Criterion

All the OLS method’s assumptions except the Jarque-Bera statistic were met. The statistical significance of each explanatory variable’s coefficient was determined by robust probabilities (*p* < 0.05), and the coefficients of each explanatory variable indicated that the strength and the direction of their association with non-uptake of DTP3 vaccination. In addition to this, there is no multicollinearity among explanatory variables of non-uptake of DTP3 vaccination because their variance inflation factor (VIF) values were less than 7.5. The overall model was significant because of statistically significant joint Wald statistic (*p* < 0.01). However, the residuals of non-uptake of DTP3 vaccination were not normally distributed across East Africa because Jarque-Bera statistic was statistically significant (*p* < 0.05). Since Koenker (BP) statistics was statistically significant, the relationships between the dependent variable (non-uptake of DTP3 vaccination) and explanatory variables were also not stationary across East Africa (there was a variation across the space) (Table 5). Therefore, a significant Koenker (BP) statistics proved that performing the MGWR could be appropriate for spatial relationships.

The MGWR model revealed that women with no maternal educational background and women with a history of fewer than four ANC visits were positively associated with non-uptake of DTP3 vaccination, whereas women who delivered in health facilities was negatively associated with non-uptake of DTP3 vaccination in East Africa. This result showed that the MGWR model explained 40.4% of variations on non-uptake of DTP3 vaccination among children aged 12–23 months in East Africa (Table 6).

**Table 6:** MGWR model summary and diagnostics of non-uptake of DTP3 vaccination among children aged 12–23 month in East Africa

Explanatory variables	Mean	Standard deviation	minimum	median	maximum	Bandwidth
intercept	0.1867	0.1299	0.0298	0.1492	0.6430	390
women with no educational background	0.1432	0.1302	-0.3474	0.1228	0.4191	462
Women who delivered in health facilities	-0.1096	0.0879	-0.3277	-0.0779	0.0215	410
Women with a history of fewer than four ANC visits	0.0690	0.0981	-0.1032	0.0505	0.3291	390
MGWR Diagnostics						
Statistics	magnitude					
Sigma- squared	0.0523					
Multiple R-square	0.418					
Adjusted R square	0.404					
AICc	-397.36					

*NB: AICc* Akaike’s Information Criterion

The OLS, GWR and MGWR models were fitted for model comparison. The Corrected Akaike Information Criterion (AICc) and adjusted R-squared (R²) performance parameters were used for model comparisons. Therefore, the MGWR model demonstrated better performance, with a lower AICc value (-397.36) and a higher adjusted R² (40.4%) compared to those of the OLS and GWR models (Table 7).

**Table 7:** Performance comparisons among OLS, GWR, and MGWR model

Spatial regression models	Performance parameter	
	Adjusted *R*-square	AICc
OLS	22.5%	677.55
GWR	39.1%	-337.68
MGWR	40.4%	-397.36

The MGWR model estimates the optimal bandwidth for each variable, allowing each covariate to operate at its own spatial scale. The optimal bandwidths were 462 for the variable women with no educational background, 410 for women who delivered in health facilities and 390 for women with a history of fewer than four ANC visits (Table 6).

**Fig. 5:**
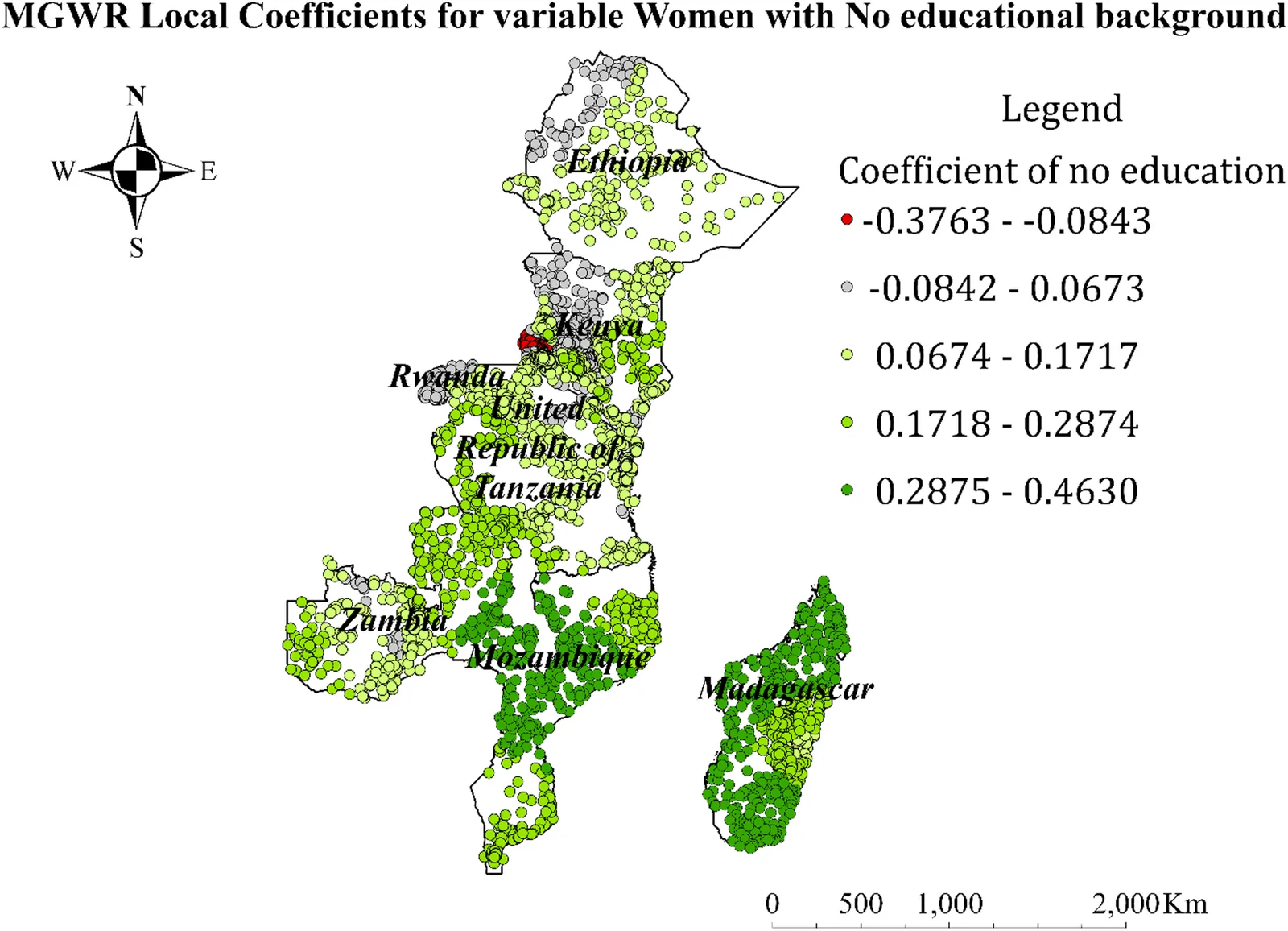
Geographically varying local coefficients for predictor No educational background of women

The MGWR result revealed that there was a positive association between women with no educational background and non-uptake of DTP3 vaccination, with coefficients ranging from (0.0674) to (0.4630). A weak positive association was observed in central, southern, and eastern parts of Ethiopia; eastern and southern parts of Kenya; northeastern and southern parts of Mozambique; the east-central regions of Madagascar; central and eastern parts of Tanzania; and southern parts of Zambia. In addition, a strong positive association between women with no educational background and non-uptake of DTP3 vaccination was observed in central Mozambique and the northern and southern parts of Madagascar. Conversely, no significant association was observed in Rwanda, northwestern parts of Ethiopia, and western parts of Kenya (Fig. 5).

**Fig. 6:**
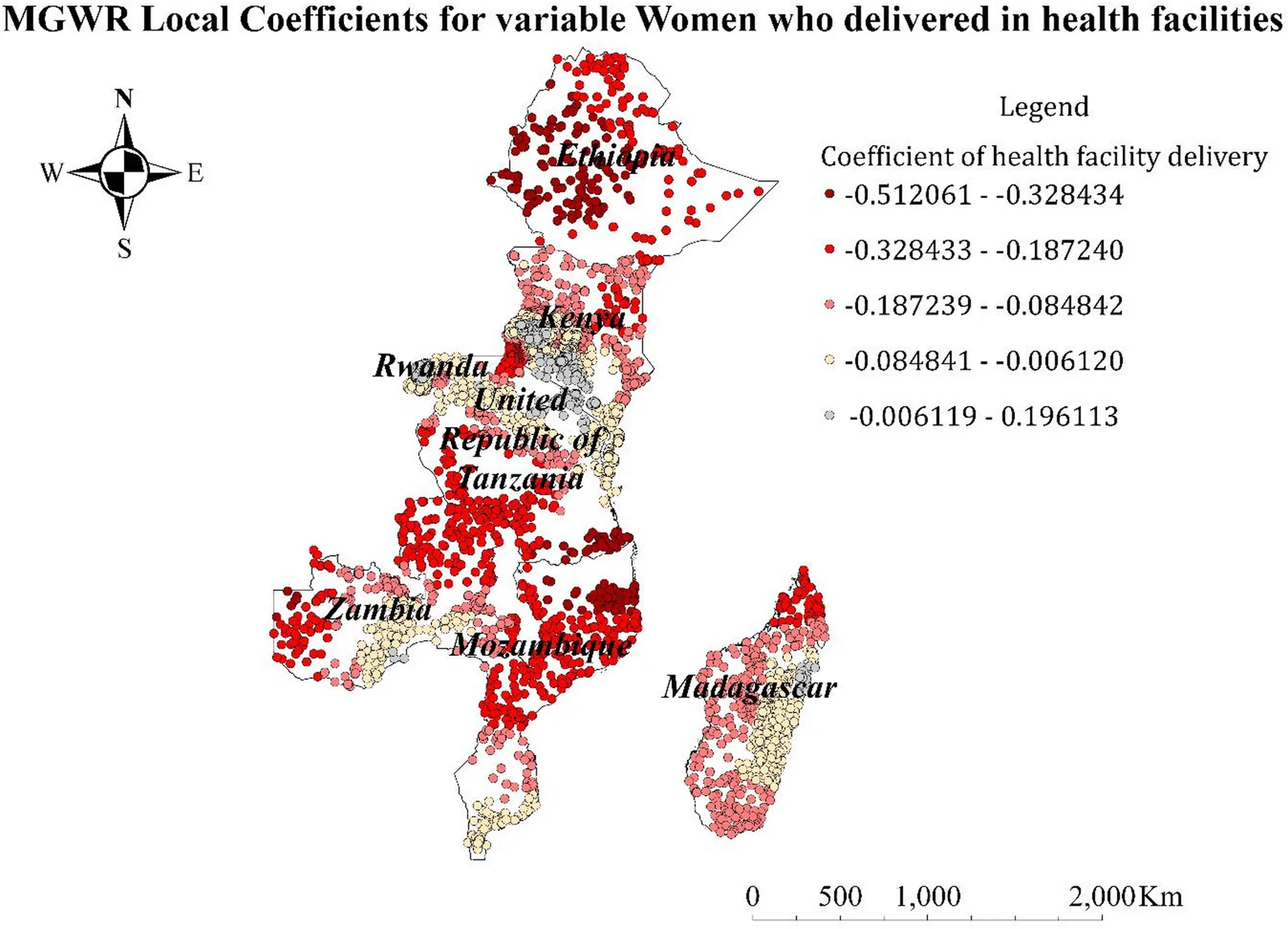
Geographically varying local coefficients for predictor women who delivered in health facilities

The MGWR analysis found a statistically significant negative relationship between women who delivered in health facilities and non-uptake of DTP3 vaccination among children aged 12–23 months, with coefficients ranging from (-0.51206) to (-0.00612). Negative association with the highest coefficient was observed in Ethiopia; the northern and eastern regions of Kenya; the central and southern parts of Tanzania; the northern and west-southern regions of Zambia; the central and northern regions of Mozambique; and the northern, southern, and east-central regions of Madagascar (Fig. 6).

**Fig. 7:**
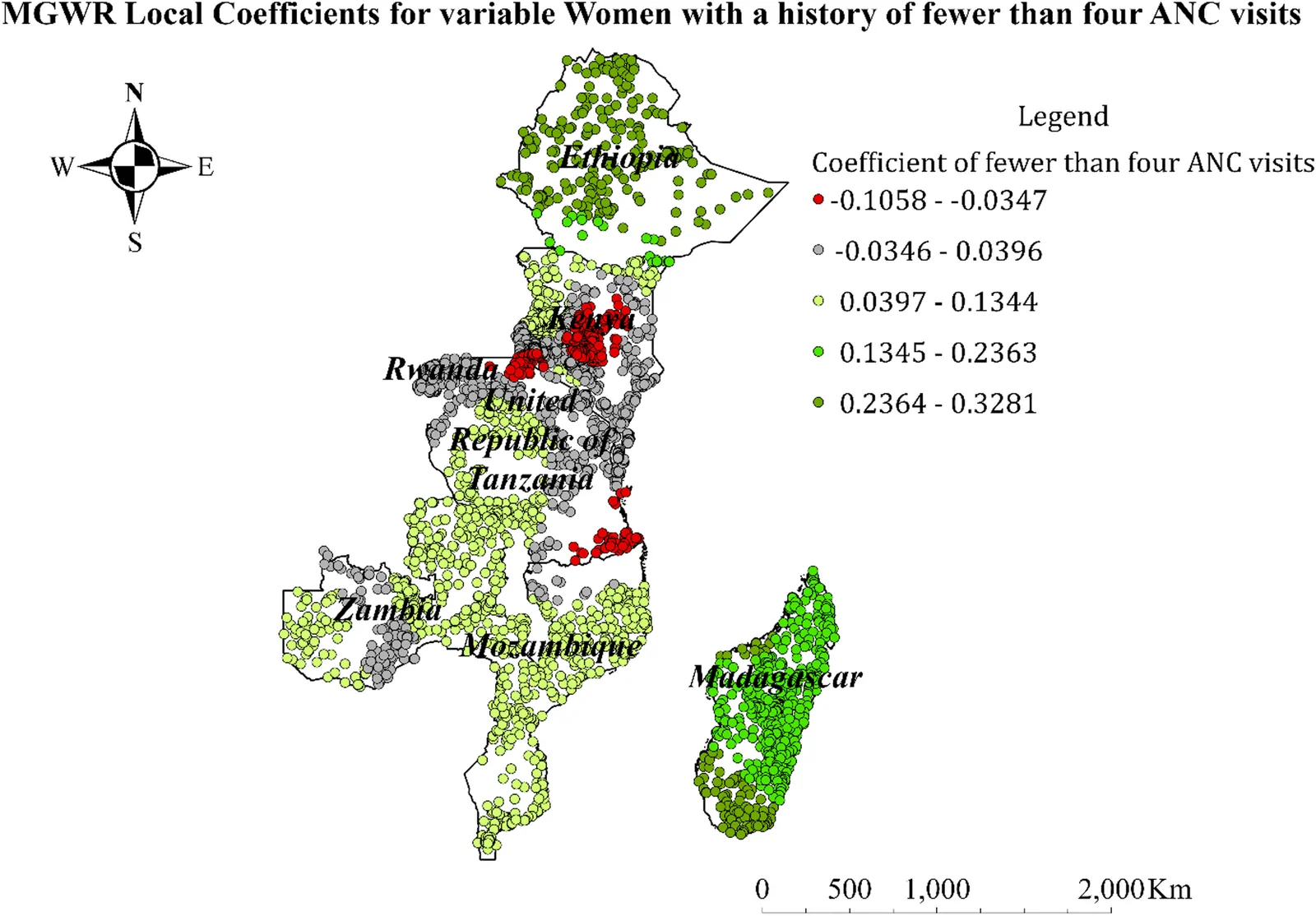
Geographically varying local coefficient for predictor women with a history of fewer than four ANC visits

The MGWR analysis result also revealed both statistically significant (positive and negative) and non-significant associations between women with a history of fewer than four ANC visits and non-uptake of DTP3 vaccination. A statistically significant positive association with coefficients varying from (0.0397) to (0.3281) was observed in Ethiopia; northern Kenya; western and southern regions of Tanzania; western and eastern parts of Zambia; Madagascar; and Mozambique. In addition, no significant association was observed in southern Kenya, Rwanda, northeastern regions of Tanzania, and west-central parts of Zambia. Unexpectedly, the negative association with coefficients ranging from (-0.1058) to (-0.0347) was observed in the southeastern regions of Tanzania and the central and southwestern regions of Kenya (Fig. 7).

## Discussion

DTP3 vaccination coverage is globally recognized as one of the key indicators to measure the overall immunization program performance. Besides, completely taking the three doses of DTP significantly reduces the risk of developing diphtheria, tetanus, and pertussis disease and the severe complication in children. Therefore, this study aimed to assess the spatial distribution and associated factors of non-uptake of DTP3 vaccination among children aged 12–23 months in East Africa.

Global Moran’s I statistics revealed that the spatial distribution of non-uptake of DTP3 vaccination in East Africa was clustered with Moran’s I value of 0.3415 and a p-value of less than 0.01. Some reasonable factors for the presence of geographical variation might be due to community-level factors. For instance, nearby residents may share quality health services, geographical barriers, cultural norms, and socioeconomic factors [33]. Getis-Ord Gi analysis detected statistically significant hotspot areas of non-uptake of DTP3 vaccination in eastern and northeastern parts of Kenya; central and northern parts of Mozambique; northern and southern parts of Madagascar; and southern and eastern parts of Ethiopia. A possible justification might be that large parts of eastern and northeastern Kenya, as well as southern and eastern parts of Ethiopia are classified as arid and semi-arid lands where access to health services is limited due to long travel distances to health facilities [34]. These areas are also predominantly inhabited by pastoralist populations whose mobility constrains access to routine immunization services. Other possible reasons include high rates of home delivery, remote nature of those areas [35], and low maternal literacy level in Northern Mozambique also contributed for the low uptake of DTP3 Vaccination. Detecting these hotspot areas inform government policy makers to prioritize resources and take targeted actions to reduce inequalities [36]. Spatial Kulldorff’s scan statistics identified a total of 291 statistically significant clusters as primary (most likely) clusters, which covers the central and most northern regions of Mozambique. In this spatial window, the log-likelihood ratio (LLR) was 597.68, and the relative risk was 3.97 at a p-value of < 0.001. This relative risk showed that children aged 12–23 months within this spatial window had a 3.97 times higher risk of having non-uptake of DTP3 vaccination than children outside this window. The detection of the estimated risk of non-uptake of DTP3 vaccination across spatial windows provide a valuable insights to guide policymakers in allocating resources and implementing targeted public health interventions [37, 38].

The MGWR analysis revealed a positive association between women with no educational background and non-uptake of DTP3 vaccination, with coefficients ranging from (0.0674) to (0.4630) observed in central, southern, and eastern parts of Ethiopia; eastern and southern parts of Kenya; northeastern and southern parts of Mozambique; the east-central regions of Madagascar; central and eastern parts of Tanzania; and southern parts of Zambia. The association was particularly strong in central Mozambique, and the northern and southern parts of Madagascar. The observed coefficients in this area indicated that the proportion of non-uptake of DTP3 vaccination among children aged 12–23 months increases by a range of 0.0674 to 0.4630% points for every 1% point increase in the proportion of uneducated women. This finding is consistence with studies done in sub Saharan Africa [39], Ethiopia [40, 41], as well as with systematic review conducted in low and middle income countries [42]. A possible justification may be that education improves women’s capacity to understand health-related information and to seek vaccination services [43]. Furthermore, educated women are more likely to have a greater awareness of the impact of immunization which enables them to make more informed decisions about their health and their children’s [44]. Therefore, community-based health education initiatives targeting women with no educational background and policies that promote female education should be strengthened to improve the uptake of DTP3 vaccination.

The MGWR analysis also revealed that the negative relationship between women who delivered in health facilities and non-uptake of DTP3 vaccination among children aged 12–23 months, with coefficients ranging from (-0.51206) to (-0.00612), was observed in Ethiopia; the northern and eastern regions of Kenya; the central and southern parts of Tanzania; the northern and west-southern regions of Zambia; the central and northern regions of Mozambique; and the northern, southern, and east-central regions of Madagascar. These coefficients in this region showed that the proportion of non-uptake of DTP3 vaccination among children aged 12–23 months decreases by a range of 0.00612 to 0.512% points for every 1% point increase in the proportion of women who delivered in health facilities. This finding is supported by other studies done in Ethiopia [40] and Tanzania [19]. The possible justification might be that Facility delivery increases mothers’ early and repeated contact with maternal and child health (MCH) services, where counseling on childhood vaccination and follow-up care provided. This continuous contact with MCH services increases the likelihood that children delivered in health facilities initiate and complete all three doses of DTP [45]. Therefore, enhancing health facility delivery and maternal and child health services should be prioritized to substantially increase the uptake of DTP3 vaccination.

The MGWR analysis also found a statistically significant positive association between women with a history of fewer than four ANC visits and non-uptake of DTP3 vaccination, with coefficients varying from (0.0397) to (0.3281), was observed in Ethiopia; northern Kenya; western and southern regions of Tanzania; western and eastern parts of Zambia; Madagascar; and Mozambique. The observed coefficients in this region showed that the proportion of non-uptake of DTP3 vaccination among children aged 12–23 months increases by a range of 0.0397 to 0.3281% points for every 1% point increase in the proportion of women with a history of fewer than four ANC visits. This finding is consistent with previous studies done in Nepal [46] and India [47]. A possible justification might be Antenatal visits provide information about the importance of completing vaccination and the growth of children. Moreover, regular contact with the health system during pregnancy ensures that mothers are well informed about the DTP vaccination [48]. Based on this finding, improving ANC attendance during pregnancy is a critical strategy to increase the uptake of DTP3 vaccination. The unexpected negative association between women with a history of fewer than four ANC visits and non-uptake of DTP3 vaccination, with coefficients varying from (-0.1058) to (-0.0347), was observed in central Kenya, northwestern and southern Tanzania. These negative coefficients indicated that DTP3 vaccine uptake among children increased even though their mothers had a history of fewer than four ANC visits. The possible justification for this unexpected results might be attributed to strong immunization outreach programs and active community engagement in vaccine promotion in Central Kenya [49]. Furthermore, In southern Tanzania, community mobilizer and health workers plays an important role to increase vaccination coverage among communities living in hard to reach areas and communities with limited access to health services [50].

## Strengths and limitations of study

This study utilized standardized and representative national DHS data from seven East African countries, which enhances the generalizability of findings. Incorporating spatial analysis techniques enabled the study to identify the areas with high non-uptake of DTP3 vaccination for targeted public health interventions. However, this study also had its own limitations. To ensure participants’ privacy and confidentiality, the DHS cluster locations were spatially displaced (up to 2 km for urban and 5 km for rural enumeration areas), which may affect the accuracy of spatial analyses. This study excluded certain East African countries due to unavailable or non-recent DHS data, which might limit the representativeness of the findings for the entire East African region. In addition, the analysis was conducted using aggregated data at the DHS cluster level, the findings may be affected by ecological fallacy and cannot be interpreted at the individual level. The study did not include supply-side variables, such as vaccine stockouts, because of the nature of the DHS data.

## Conclusions

The spatial distribution of non-uptake of DTP3 vaccination among children aged 12–23 months across east Africa was clustered. Statistically significant hotspot areas of non-uptake of DTP3 vaccination were observed in eastern and northeastern parts of Kenya; central and northern parts of Mozambique; northern and southern parts of Madagascar; and southern and eastern parts of Ethiopia. A total of 291 statistically significant clusters were identified as primary (most likely) clusters, which were observed in central and most northern regions of Mozambique. MGWR revealed that women with no maternal educational background and women with a history of fewer than four ANC visits were positively associated with non-uptake of DTP3 vaccination, whereas women who delivered in health facilities was negatively associated with non-uptake of DTP3 vaccination in East Africa. Therefore, these findings enable policymakers and concerned bodies to implement targeted public health interventions and to prioritize resources strategically to reduce health inequalities and improve the uptake of DTP3 vaccination. Moreover, researchers should adopt a qualitative approach analysis for a deeper understanding of the underlying reasons for non-uptake of DTP3 vaccination among children aged 12–23 months.

## Data Availability

The data used in this study are publicly available on Demographic and Health Surveys (DHS) website (https://dhsprogram.com/data/AccessInstructions.cfm).

## Abbreviations

DHS: Demographic Health Survey
GWR: Geographically Weighted Regression
OLS: Ordinary Least Square
MGWR: Multiscale Geographic Weighted Regression
WHO: World Health Organization

## References

[1] ChardAN, Gacic-Dobo M, Diallo MS, Sodha SV, Wallace AS. Routine Vaccination Coverage — Worldwide, 2019. MMWR Morb Mortal Wkly Rep. 2020;69:1706–10. 10.15585/mmwr.mm6945a7 PMC7660669 33187395

[2] WHO. Global immunization efforts have saved at least 154 million lives over the past 50 years. Geneva; 2024. Available from: https://www.who.int/news/item/24-04-2024-global-immunization-efforts-have-saved-at-least-154-million-lives-over-the-past-50-years.

[3] Pan American Health Organization. Diphtheria, tetanus, and pertussis (DTP) vaccine. Washington DC. Available from: https://www.paho.org/en/diphtheria-tetanus-and-pertussis-dtp-vaccine.

[4] Medicines Sans Frontiers. Diphtheria-tetanus-pertussis vaccine (DTP): Médecins Sans Frontières. 2024. Available from: https://medicalguidelines.msf.org/en/viewport/EssDr/english/diphteria-tetanus-pertussis-vaccine-dtp-16688268.html.

[5] Sodha SV, Dietz V. Strengthening routine immunization systems to improve global vaccination coverage. Br Med Bull. 2015;113(1):5–14. 25649959 10.1093/bmb/ldv001 PMC10350316

[6] WHO, Immunization Agenda. 2030: A global strategy to leave no one behind. World Health Organization; 2020. Available from: https://www.who.int/publications/m/item/immunization-agenda-2030-a-global-strategy-to-leave-no-one-behind. 10.1016/j.vaccine.2022.11.042 39004466

[7] WHO. Immunization coverage. Geneva; 2025. Available from: https://www.who.int/news-room/fact-sheets/detail/immunization-coverage.

[8] UNICEF. Immunization New York: United Nations Children’s Fund. 2025. Available from: https://data.unicef.org/topic/child-health/immunization/.

[9] Gavi The Vaccine Alliance. Geneva; 2025. Available from: https://www.gavi.org/news/media-room/2024-wuenic-immunisation-lower-income-countries.

[10] AfricaCDC, Communiqué. Continental Immunization Strategy Adoption. 2026. Available from: https://africacdc.org/news-item/communique-continental-immunization-strategy-adoption/.

[11] (CDC) CfDCaP. Vaccines & Immunizations: DTaP (Diphtheria, Tetanus, Pertussis) Vaccine VIS. 2021. Available from: https://www.cdc.gov/vaccines/hcp/current-vis/dtap.html.

[12] CDC. Clinical Features of Diphtheria: U.S. Department of Health and Human Services. 2025. Available from: https://www.cdc.gov/diphtheria/hcp/clinical-signs/index.html.

[13] Birch TB, Bleck TP. Tetanus (Clostridium tetani). In Mandell, Douglas, and Bennett's Principles and Practice of Infectious Diseases, 9th Edition: Volume 1-2. Elsevier; 2019. p. 2948-53. e2.

[14] George EK, De Jesus O, Tobin EH, Vivekanandan R. Tetanus (Clostridium tetani infection). StatPearls [Internet]: StatPearls Publishing; 2024. 29494091

[15] Wang C, Zhang H, Zhang Y, Xu L, Miao M, Yang H, et al. Analysis of clinical characteristics of severe pertussis in infants and children: a retrospective study. BMC Pediatr. 2021;21(1):65. 33546645 10.1186/s12887-021-02507-4 PMC7863367

[16] CDC. Clinical Features of Pertussis: U.S. Department of Health and Human Services. 2025. Available from: https://www.cdc.gov/pertussis/hcp/clinical-signs/index.html.

[17] Holipah H, Maharani A, Sujarwoto S, Hinoura T, Kuroda Y, Trends. Spatial Disparities, and Social Determinants of DTP3 Immunization Status in Indonesia 2004–2016. Vaccines. 2020;8(3):518. 32927862 10.3390/vaccines8030518 PMC7563731

[18] De Figueiredo A, Johnston IG, Smith DM, Agarwal S, Larson HJ, Jones NS. Forecasted trends in vaccination coverage and correlations with socioeconomic factors: a global time-series analysis over 30 years. Lancet Global Health. 2016;4(10):e726–35. 27569362 10.1016/S2214-109X(16)30167-X

[19] Nadella P, Smith ER, Muhihi A, Noor RA, Masanja H, Fawzi WW, et al. Determinants of delayed or incomplete diphtheria-tetanus-pertussis vaccination in parallel urban and rural birth cohorts of 30,956 infants in Tanzania. BMC Infect Dis. 2019;19(1):188. 30808282 10.1186/s12879-019-3828-3 PMC6390320

[20] Adetokunboh OO, Uthman OA, Wiysonge CS. Non-uptake of childhood vaccination among the children of HIV-infected mothers in sub-Saharan Africa: A multilevel analysis. Hum Vaccines Immunotherapeutics. 2018;14(10):2405–13. 10.1080/21645515.2018.1502524 PMC6290935 30036129

[21] Lanza-León P, Cantarero-Prieto D, Pascual-Sáez M. Exploring trends and determinants of basic childhood vaccination coverage: Empirical evidence over 41 years. PLoS ONE. 2024;19(3):e0300404. 38512892 10.1371/journal.pone.0300404 PMC10956826

[22] Usman HR, Kristensen S, Rahbar MH, Vermund SH, Habib F, Chamot E. Determinants of third dose of diphtheria-tetanus-pertussis (DTP) completion among children who received DTP1 at rural immunization centres in Pakistan: a cohort study. Trop Med Int Health. 2010;15(1):140–7. 19930140 10.1111/j.1365-3156.2009.02432.x PMC2858790

[23] Araújo Veras AAC, Arruda Vidal S, Costa de Macêdo V, de Carvalho Lima M, Cabral de Lira PI, da Fonseca Lima EJ, Batista Filho M. Prevalence, Trends and Conditions for the DTP3 Vaccine: A 25-Year Historical Perspective. Risk Manag Healthc Policy. 2021;14:4301–10 10.2147/RMHP.S312263 10.2147/RMHP.S312263 PMC8524252 34703341

[24] Krishnamoorthy Y, Rehman T. Impact of antenatal care visits on childhood immunization: a propensity score-matched analysis using nationally representative survey. Fam Pract. 2021;39(4):603–9. 10.1093/fampra/cmab124 34564727

[25] world population review. East african countries. 2025. Available from: https://worldpopulationreview.com/country-rankings/east-african-countries.

[26] United Nation. African Studies and African Country Resources@ Pitt. East African Countries. 2023.

[27] Croft TN, Marshall AM, Allen CK, Arnold F, Assaf S, Balian S. Guide to DHS statistics. Rockville: ICF. 2018;645:292–303.

[28] BeaulieuA, Ducharme J, Thibeault C, Akani BC, Ziegler D, Hogan D, et al. The Immunization Agenda 2030 Strategy to reach zero-dose children in low-income and middle-income countries: a scoping review. BMJ Global Health. 2025;10(8):e018293. 10.1136/bmjgh-2024-018293 10.1136/bmjgh-2024-018293 PMC12336475 40763981

[29] Tsai P-J, Lin M-L, Chu C-M, Perng C-H. Spatial autocorrelation analysis of health care hotspots in Taiwan in 2006. BMC Public Health. 2009;9:1–13. 20003460 10.1186/1471-2458-9-464 PMC2799414

[30] Wulder M, Boots B. Local spatial autocorrelation characteristics of remotely sensed imagery assessed with the Getis statistic. Int J Remote Sens. 1998;19(11):2223–31.

[31] Kulldorff M. A Spatial Scan Statistic. Commun Stat - Theory Methods. 1997;26:1481–96.

[32] Fotheringham AS, Brunsdon C, Charlton M. Geographically Weighted Regression: The Analysis of Spatially Varying Relationships. John Wiley & Sons; 2003. ISBN: 9780470855256.

[33] UNFPA. The Social Determinants of Maternal Death and Disability. UNFPA; 2012. https://www.unfpa.org/resources/social-determinants-maternal-death-and-disability

[34] Joseph NK, Macharia PM, Ouma PO, Mumo J, Jalang’o R, Wagacha PW, et al. Spatial access inequities and childhood immunisation uptake in Kenya. BMC Public Health. 2020;20(1):1407. 32933501 10.1186/s12889-020-09486-8 PMC7493983

[35] Jean Simon D, Kondo Tokpovi VC, Dianou K, Okonji OC, Kiragu A, Olorunsaiye CZ, et al. Regional, subregional and country-level full vaccination coverage in children aged 12–23 months for 34 countries in sub-Saharan Africa: a global analysis using demographic and health survey data. BMJ Global Health. 2025;10(3):e018333. 40090697 10.1136/bmjgh-2024-018333 PMC11911682

[36] BarthelM, Fava JA, Harnanan CA, Strothmann P, Khan S, Miller S. Hotspots analysis: providing the focus for action. In Life cycle management. Springer Netherlands; 2015. p. 149–67. 10.1007/978-94-017-7221-1_12

[37] Aboushady AT, Mansour F, El Maghraby M, Teixeira B, Cunha S, Dantas MM, et al. The use of SatScan software to map spatiotemporal trends and detect disease clusters: a systematic review. Commun Med (Lond). 2025;5(1):82. 40119121 10.1038/s43856-024-00699-1 PMC11928592

[38] Azage M, Kumie A, Worku A, Bagtzoglou AC. Childhood Diarrhea Exhibits Spatiotemporal Variation in Northwest Ethiopia: A SaTScan Spatial Statistical Analysis. PLoS ONE. 2015;10(12):e0144690. 26690058 10.1371/journal.pone.0144690 PMC4687002

[39] Adetokunboh OO, Uthman OA, Wiysonge CS. Non-uptake of childhood vaccination among the children of HIV-infected mothers in sub-Saharan Africa: A multilevel analysis. Hum Vaccin Immunother. 2018;14(10):2405–13. 30036129 10.1080/21645515.2018.1502524 PMC6290935

[40] Fekadu H, Mekonnen W, Adugna A, Kloos H, Hailemariam D. Trends of inequality in DPT3 immunization services utilization in Ethiopia and its determinant factors: Evidence from Ethiopian demographic and health surveys, 2000–2019. PLoS ONE. 2024;19(1):e0293337. 38227594 10.1371/journal.pone.0293337 PMC10791004

[41] Nour TY, Farah AM, Ali OM, Osman MO, Aden MA, Abate KH. Predictors of immunization coverage among 12–23 month old children in Ethiopia: systematic review and meta-analysis. BMC Public Health. 2020;20(1):1803. 33243208 10.1186/s12889-020-09890-0 PMC7689978

[42] Ali HA, Hartner A-M, Echeverria-Londono S, Roth J, Li X, Abbas K, et al. Vaccine equity in low and middle income countries: a systematic review and meta-analysis. Int J Equity Health. 2022;21(1):82. 35701823 10.1186/s12939-022-01678-5 PMC9194352

[43] Johri M, Subramanian S, Sylvestre M-P, Dudeja S, Chandra D, Koné GK, et al. Association between maternal health literacy and child vaccination in India: a cross-sectional study. J Epidemiol Community Health. 2015;69(9):849–57. 25827469 10.1136/jech-2014-205436 PMC4552929

[44] Mehata S, Paudel YR, Dariang M, Aryal KK, Lal BK, Khanal MN, et al. Trends and inequalities in use of maternal health care services in Nepal: strategy in the search for improvements. Biomed Res Int. 2017;2017(1):5079234. 28808658 10.1155/2017/5079234 PMC5541802

[45] Fatiregun AA, Etukiren EE. Determinants of uptake of third doses of oral polio and DTP vaccines in the Ibadan North Local Government Area of Nigeria. Int Health. 2014;6(3):213–24. 24844557 10.1093/inthealth/ihu027

[46] Thapa K, Adhikary P, Faruquee MH, Suwal BR. Associated Factors for Dropout of First Vs Third Doses of Diphtheria Tetanus Pertussis (DPT) Vaccination in Nepal. Adv Prev Med. 2021;2021:1319090. 33959398 10.1155/2021/1319090 PMC8075685

[47] Dhalaria P, Kapur S, Singh AK, Priyadarshini P, Dutta M, Arora H, et al. Exploring the Pattern of Immunization Dropout among Children in India: A District-Level Comparative Analysis. Vaccines (Basel). 2023;11(4):836. 10.3390/vaccines11040836 10.3390/vaccines11040836 PMC10143302 37112748

[48] Mutabazi D, Ruganintwari B, de Dieu Iradukunda J, Ruton H. Assessing the Continuum of Maternal and Child Health. Correlations Between ANC Indicators and Immunization Coverage in Rwanda. 2025.

[49] Institute SV. Finding new ways to engage communities in vaccinations in Kenya: Gavi, the Vaccine Alliance. 2024. Available from: https://www.gavi.org/vaccineswork/finding-new-effective-ways-engage-communities-life-saving-vaccinations-kenya.

[50] Africa WROf. Vaccination in the wild: Tanzania’s vaccination campaign leaves no one behind: WHO Regional Office for Africa. 2024. Available from: https://www.afro.who.int/photo-story/vaccination-wild-tanzanias-vaccination-campaign-leaves-no-one-behind.

